# A causal inference explanation for enhancement of multisensory integration by co-articulation

**DOI:** 10.1038/s41598-018-36772-8

**Published:** 2018-12-21

**Authors:** John F. Magnotti, Kristen B. Smith, Marcelo Salinas, Jacqunae Mays, Lin L. Zhu, Michael S. Beauchamp

**Affiliations:** 1 Department of Neurosurgery, Baylor College of Medicine, Houston, TX USA; 2 Department of Neuroscience, Baylor College of Medicine, Houston, TX USA

## Abstract

The McGurk effect is a popular assay of multisensory integration in which participants report the illusory percept of “da” when presented with incongruent auditory “ba” and visual “ga” (AbaVga). While the original publication describing the effect found that 98% of participants perceived it, later studies reported much lower prevalence, ranging from 17% to 81%. Understanding the source of this variability is important for interpreting the panoply of studies that examine McGurk prevalence between groups, including clinical populations such as individuals with autism or schizophrenia. The original publication used stimuli consisting of multiple repetitions of a co-articulated syllable (three repetitions, AgagaVbaba). Later studies used stimuli without repetition or co-articulation (AbaVga) and used congruent syllables from the same talker as a control. In three experiments, we tested how stimulus repetition, co-articulation, and talker repetition affect McGurk prevalence. Repetition with co-articulation increased prevalence by 20%, while repetition without co-articulation and talker repetition had no effect. A fourth experiment compared the effect of the on-line testing used in the first three experiments with the in-person testing used in the original publication; no differences were observed. We interpret our results in the framework of causal inference: co-articulation increases the evidence that auditory and visual speech tokens arise from the same talker, increasing tolerance for content disparity and likelihood of integration. The results provide a principled explanation for how co-articulation aids multisensory integration and can explain the high prevalence of the McGurk effect in the initial publication.

## Introduction

In multisensory speech perception, integrating visual mouth movements with auditory speech improves perceptual accuracy, but only if the information in the modalities arises from the same talker. Causal inference provides a theoretical framework for quantifying this determination^1–4^ and is especially informative when conflict between modalities is experimentally introduced^5–7^. A well-known example of cue conflict in audiovisual speech is the McGurk effect. If auditory “ba” is paired with visual “ga”, the two conflicting syllables are integrated to form the percept “da”^8^. In their original report, McGurk and MacDonald reported that the illusion was perceived by 98% of adults. In contrast, estimates of McGurk prevalence in recent studies are highly variable but are all considerably lower than the original report, ranging from 17% to 81% of adults^9–12^.

Understanding the factors that contribute to McGurk prevalence are important for two main reasons. First, the illusion is an important tool for studying multisensory speech perception, including the underlying brain mechanisms^13–16^, computational models^17–19^, and perceptual phenomenology^20–23^. Second, the McGurk effect is used as an assay of multisensory integration in a wide range of clinical populations, including children with autism spectrum disorders^24–26^, adults with schizophrenia^27,28^, and cochlear implant users^29,30^. Invariably, these studies compare the clinical population to healthy or typically-developing controls, assuming that the variability within each group will be overcome by the between-group difference. Understanding the factors that contribute to McGurk prevalence will allow us to discern which sources of variance are caused by individual/group-level variation and which are caused by differences in experimental procedures (*e*.*g*., testing setup, stimuli).

Since the stimuli used in the original study are not available^31^, subsequent studies used stimuli recorded from different talkers under different conditions. A comparison of these stimuli showed that they differ widely in efficacy^10,11^. However, this does not explain why modern stimuli are consistently *weaker* than the stimuli used in the original study.

A comparison of the original and later stimuli provides a possible answer. In the original report, the stimulus consisted of three presentations of a double-syllable (*e*.*g*. “ba-ba, ba-ba, ba-ba” dubbed over “ga-ga, ga-ga, ga-ga”) while later studies typically use a single syllable repeated once (*e*.*g*. “ba” dubbed over “ga”). From the vantage point of causal inference, there are three important differences between the original and modern studies. First, double syllables contain co-articulation: when syllables are spoken in sequence in a word, the manner of pronunciation varies compared to a syllable uttered in isolation. This co-articulation changes the expected lip/voice correspondence and might be expected to mask the discrepancy between the auditory and visual syllables, increasing stimulus strength. Second, repeated presentations of a syllable within one stimulus provides more opportunity to extract the visual speech information, increasing the relative influence of visual speech on auditory speech perception, the key ingredient in the McGurk effect. Third, modern studies often use the same talker repeated across multiple stimuli, including congruent control stimuli. Repeated exposure to a talker may make the observer more familiar with the talker’s natural speech gestures. This would make the incongruence in the McGurk stimuli more apparent and render them less effective. One previous study assessed talker familiarity on McGurk perception, with talkers familiar to some of the participants but not to others^32^. This study did not find a significant difference between familiar and unfamiliar talkers for an AbaVga stimulus similar to that used in the present study. However, this study employed a between-subjects design and did not specifically emphasize familiarity with a talker’s syllable articulation.

This multiplicity of factors with the potential to influence McGurk prevalence motivated a series of experiment. In experiment 1, McGurk prevalence for single syllables repeated once (similar to most modern stimuli) was compared with double-syllables with co-articulation repeated three times (similar to the original stimuli). Experiment 2 tested the effect of syllable repetition without co-articulation, while experiment 3 tested the effect of talker familiarity. Each of these experiments used within-subject experimental designs to maximize statistical power. Because an online testing service (Amazon Mechanical Turk) was used to conduct experiments 1–3, experiment 4 compared online and in-person setups for studying the McGurk effect.

## Results

### Experiment 1: Repetition with Co-Articulation Increases McGurk Prevalence

In experiment 1, 76 participants reported their percept of two different stimulus types. The first stimulus type was modeled after the most common stimulus in current studies of the McGurk effect: a single repetition of an incongruent audiovisual syllable (auditory component “ba”, visual component “ga”). The second stimulus type, modeled after the original McGurk stimulus, consisted of a double syllable (providing co-articulation) repeated three times for six total repetitions (auditory component “ba-ba, ba-ba, ba-ba”, visual component “ga-ga, ga-ga, ga-ga”).

**Figure 1:**
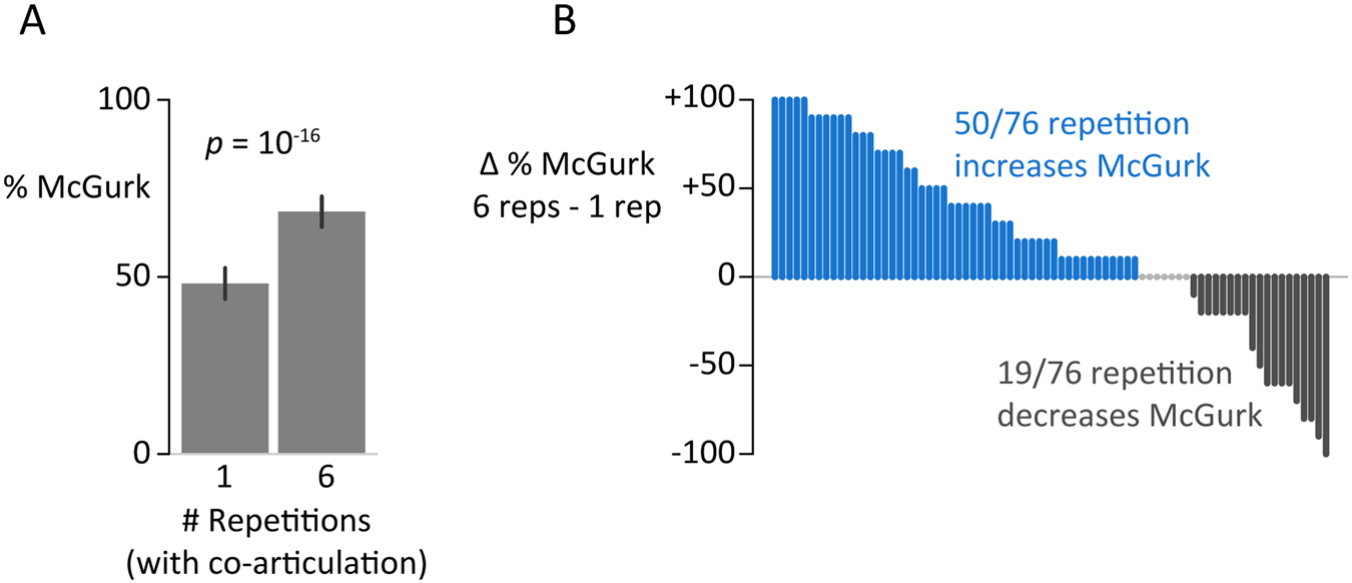
Significant effect of syllable repetition with co-articulation. (**A**) Percent of trials on which participants reported the McGurk percept of “da” for the combination of the auditory syllable “ba” dubbed over the visual syllable “ga”, AbaVga. The syllable was repeated either one time or six times with co-articulation (AbaVga *vs*. AbabaVgaga, AbabaVgaga, AbabaVgaga). Mean across all trials and participants, error bars show standard error of the mean, *p*-value from the categorical repetition parameter of the GLMM. (**B**) Individual participant data for the difference between six repetitions and one repetition, sorted by difference. One bar per participant, blue bars show participants with increased McGurk responses for six repetitions, gray circles show participants with no change, black bars show participants with decreased McGurk responses.

As shown in Fig. 1A, there was a significant difference between the one-repetition and six-repetition stimuli. The six-repetition stimuli had a higher McGurk prevalence (mean = 68% ± 4%, standard error of the mean across participants) than the non-repeated single syllable (48% ± 4%), significantly different by paired *t*-test, *t*(75) = 3.5, *p* = 0.0009. However, *t-*tests are poorly suited to proportional data such as McGurk prevalence. For instance, the *t*-test considers increases in McGurk prevalence from 0.1 to 0.2 or from 0.5 to 0.6 as identical even though they represent different effect sizes: 0.1 to 0.2 is an increase of 225% in the odds-ratio (0.1/0.9 ÷ 0.2/0.8) while 0.5 to 0.6 is an increase of only 150% (0.6/0.4 ÷ 0.5/0.5). Therefore, we performed an additional analysis using generalized linear mixed-effects models (GLMM). Comparing the goodness of fit of different models using the Bayesian Information Criterion (BIC) allows for an assessment of their relative predictive validity while avoiding the problems that arise from selecting an arbitrary statistical significance threshold and using it to reject (or fail to reject) a null hypothesis. Two competing models were constructed, one of which included a fixed effect of repetition (both models included participant and stimulus as random effects and stimulus counterbalancing group as a fixed effect). Model comparison decisively favored the model that included repetition (ΔBIC = 73, model with repetition factor is 10^17^ times more likely) and yielded an estimated odds-ratio for the repetition effect of 3.1 ± 1.1 (standard error of the estimate; *z* = 8.7, *p* = 10^−18^). Translating the odds-ratio into McGurk prevalence, an average participant would be expected to increase from 49% McGurk percepts for the one-repetition stimuli to 74% McGurk for the six-repetition stimuli (prediction obtained by marginalizing over all other model factors).

To avoid any carryover effects, subjects heard different talkers speaking the one-repetition and six-repetition stimuli. To assess if the repetition effect interacted with talker identity, we constructed another GLMM that allowed for differences in the repetition effect between talkers, but this did not improve the model fit (ΔBIC = 10 in favor of the simpler model).

During the same testing session, participants viewed a third single-syllable McGurk stimulus recorded from a different talker (each subject viewed only one stimulus from each talker). This control stimulus evoked similar rates of McGurk percepts in the current study (73% ± 4%) as in an earlier study that used the same stimulus (75% ± 3%; independent *t*-test: *t*(191) = 0.5, *p* = 0.6) (S2.5 from^11^) demonstrating that the repetition manipulation *increased* illusory percepts for the repeated stimulus rather than *decreasing* them for non-repeated stimuli.

Although the majority of participants (50/76) experienced an increase in McGurk percepts for the repeated stimulus, there was substantial inter-individual variability in the strength and direction of the repetition effect (Fig. 1B).

### Experiment 2: Repetition without co-articulation does not change McGurk prevalence

In experiment 1, the six-repetition stimuli contained co-articulation within each double syllable. To separate the effect of co-articulation from repetition alone, in experiment 2 we constructed stimuli consisting of eight talkers speaking one-syllable McGurk stimuli, repeated one, two, three or six times. These new stimuli contained multiple repetitions *without* co-articulation (*e*.*g*., repeated “ba, ba” instead of a continuous speech stream “ba-ba”).

**Figure 2:**
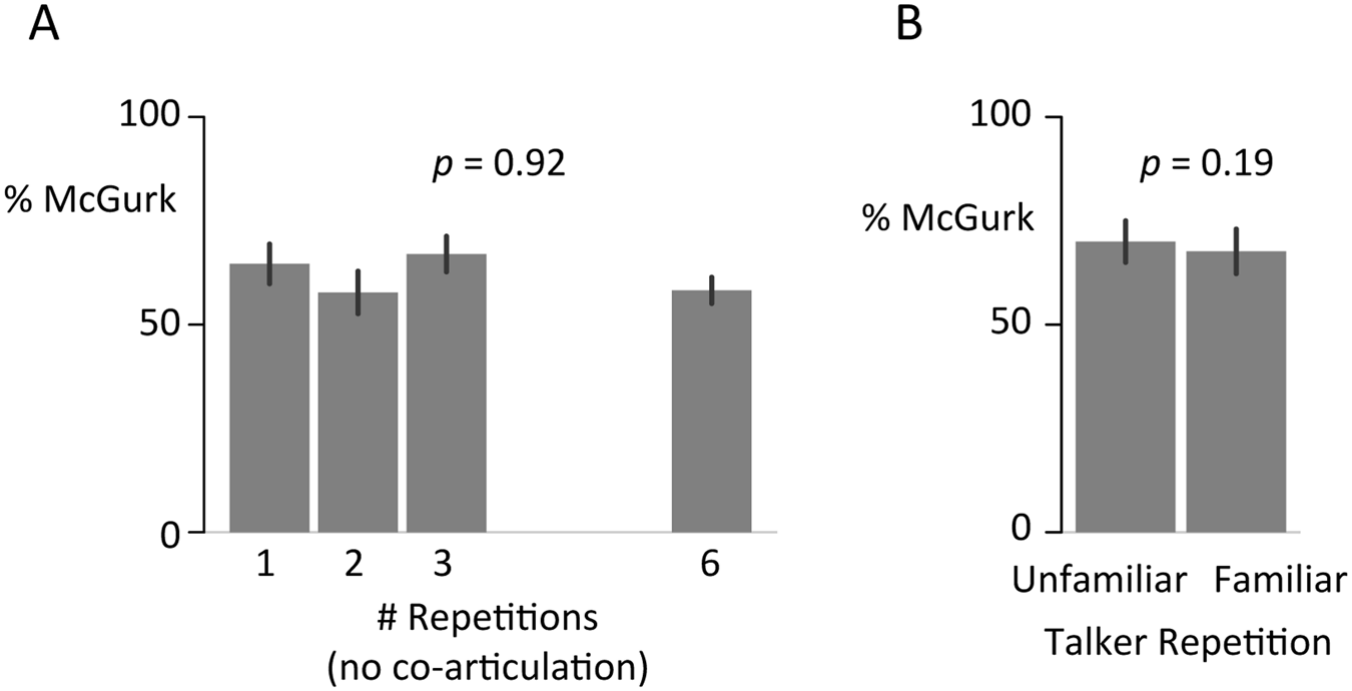
(**A**) No effect for syllable repetition without co-articulation. Percent of trials on which participants reported the McGurk percept of “da” for AbaVga repeated 1x, 2x, 3x, or 6x without co-articulation. Mean across all trials and participants, error bars show standard error of the mean, *p*-value from the scalar repetition parameter of the GLMM. (**B**) No effect for talker repetition. McGurk percentage for an unfamiliar talker or a talker familiarized with a pre-exposure manipulation. *P*-value from the categorical familiarity parameter of the GLMM.

Figure 2A shows the results from 40 participants viewing stimuli containing repetition without co-articulation. McGurk rates were similar across the different numbers of repetitions (65% ± 5% for 1-rep, 58% ± 5% for 2-rep, 67% ± 4% for 3-rep, and 57% ± 3% for 6-rep). To quantify this lack of difference, we compared two GLMMs with and without fixed effects for repetition. Unlike in experiment 1, the model *without* the fixed effect of repetition fit the data better (ΔBIC = 6 in favor of the simpler model; *p*-value for repetition parameter = 0.921; repetition effect odds-ratio = 0.98 ± 1.18).

Although repetition did not increase the prevalence of the McGurk effect, it might still impact perception by decreasing the variability of an individual’s percept. If individuals are treating each presentation as a repetition of an identical exemplar, they should get more precise information from repeated presentations than from a single presentation. This increase in precision is akin to the increase in precision produced by multisensory integration in general. To test for a change in variance, we first converted proportion “da” to a variance term (binomial variance = n*p*q, where *n* is the number of trials, *p* the proportion of McGurk responses, and *q* the proportion of non-McGurk responses). Next, we fit linear mixed effects models (not GLMMs) with and without a linear term for number of repetitions (both models had random effects for participant and stimulus). The model without the repetition parameter fit the data better (ΔBIC = 6 in favor of the simpler model; *p*-value for repetition parameter = 0.926; repetition effect parameter = −0.003 ± 0.03), suggesting no change in variance for different numbers of stimulus repetitions.

### Experiment 3: Repetition of Talker Does Not Change McGurk Prevalence

In experiment 3, we attempted to modify the weight given to the lip/voice correspondence cue by familiarizing participants with exemplars of a given talker’s congruent audiovisual speech stimuli (audiovisual “ba”, “da”, and “ga”). After familiarization with the congruent stimuli, the participant then viewed McGurk stimuli from the familiar talker and an unfamiliar talker. Stimuli from two different talkers were used with the familiar and unfamiliar talkers counterbalanced across participants to control for any talker-specific effects.

As shown in Fig. 2B, participants reported similar rates of McGurk effect for familiar and unfamiliar talkers: 70% ± 5% for familiar, 68% ± 5% for unfamiliar, *t*(54) = 0.7, *p* = 0.48. We compared two GLMMs with and without a fixed effect of talker familiarity (both models had random effects of talker and participant). The model *without* the effect of talker familiarity fit the data better (ΔBIC = 3 in favor of the simpler model; *p*-value for familiarity parameter = 0.19; familiarity effect odds-ratio = 0.77 ± 1.2). Although the odds ratio is in the predicted direction (familiarity with a talker reduces McGurk prevalence), the data suggest brief familiarization does not have a robust effect on integration.

### Experiment 4: On-line vs. in-person testing does not change McGurk prevalence

**Figure 3:**
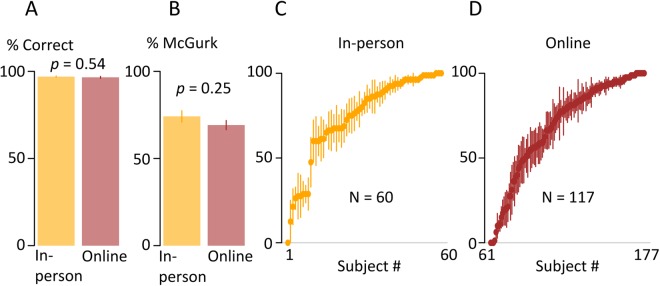
No difference between in-person and online testing. (**A**) Accuracy of responses for auditory-only syllables, mean and standard error within in-person (orange) and online (red) participants, *p*-value from *t*-test. (**B**) Percent of McGurk responses averaged within in-person and online participants. (**C**) Individual participant McGurk prevalence for in-person participants. One symbol per participant, sorted by magnitude, error bars show standard error across stimuli. (**D**) Individual participant McGurk prevalence for online participants.

To assess the generalizability of the results obtained using on-line testing in experiments 1–3, in experiment 4 we compared data collected using identical testing procedures and stimuli from 60 participants tested in-person with 160 participants tested on-line. As shown in Fig. 3A, both groups were near ceiling accuracy for discriminating control stimuli consisting of auditory-only syllables: 97% ± 1% in-person vs. 97% ± 1% on-line, *t*(108) = 0.61, *p* = 0.54).

Previous studies have described tremendous variability across individuals in their susceptibility to the McGurk effect, a finding that was replicated in both in-person and online samples (Fig. 3C,D). In both groups, the complete range of McGurk susceptibility was observed: within each group, some participants never reported the McGurk percept on any presentation of any stimulus, while some participants reported the McGurk percept on every presentation of every stimulus. Rates of McGurk perception similar between groups (Fig. 3B; in-person, mean = 74%, sd = 26% *vs*. online, mean = 69%, sd = 29%; *t*(176)−1.14, *p* = 0.26]. To quantify this effect, GLMMs were compared with and without a factor for testing mechanism (in-person *vs*. online). Model comparison favored the model that did not include a factor for testing mechanism (ΔBIC of 6, estimated odds-ratio = 0.65, *p* = 0.2).

## Discussion

There is a large difference in McGurk prevalence between the initial publication describing the illusion (98%; McGurk & MacDonald, 1976) and subsequent studies (from 18% to 81% in one review; Magnotti & Beauchamp, 2015). Since the stimuli used in the original publication are no longer available^31^, subsequent studies used stimuli recorded from different talkers. While these stimuli differ widely in efficacy^10,11^ this does not explain why modern stimuli are consistently *weaker* than the original stimuli. We found that McGurk syllables repeated with co-articulation, as used in the initial publication, reliably evoke more McGurk percepts than single syllables, as used in most subsequent studies. This boost (a tripling in the probability of perceiving the illusion for an average individual, leading to a net increase of 20% at the group level) explains a significant fraction of the difference in prevalence in the McGurk effect between the original publication and subsequent studies.

**Figure 4:**
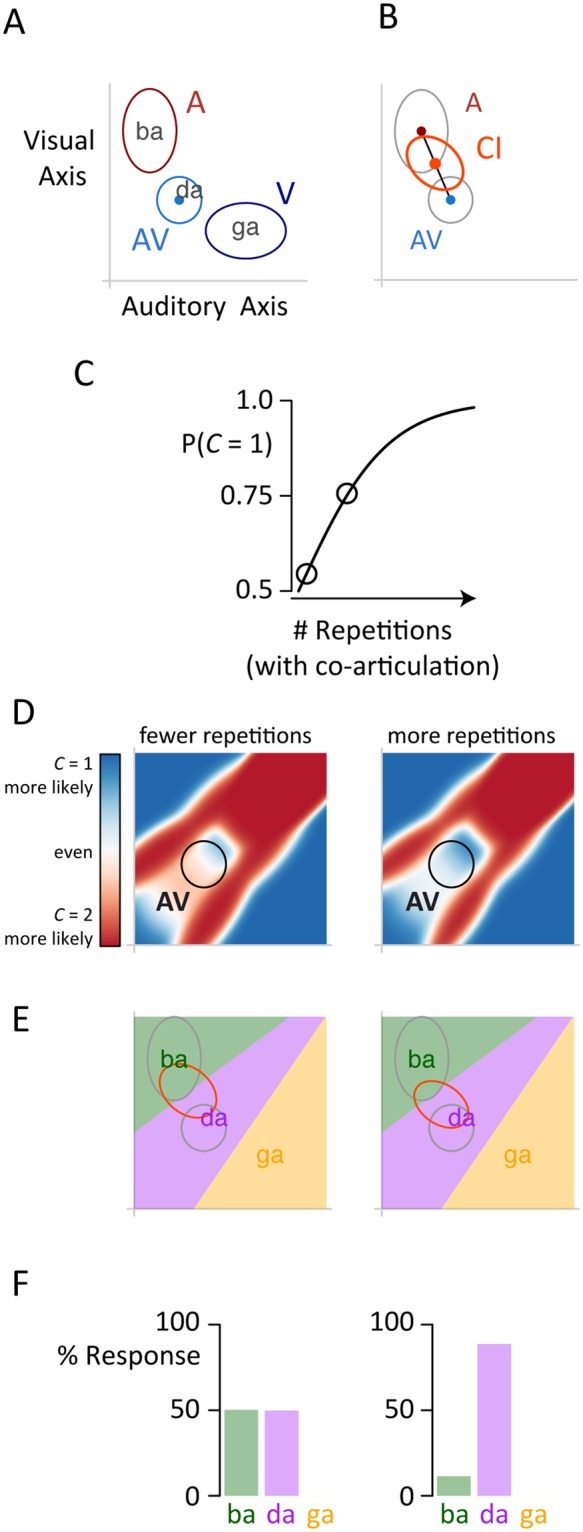
Causal inference explanation of repetition with co-articulation. (**A**) A two-dimensional representational space is constructed with auditory speech features on the *x*-axis and visual speech features on the *y*-axis. Three syllables are placed in this space according to their feature values. When presented with an auditory “ba”, the participant encodes the feature values with noise (red ellipse labeled “A” shows the sampling distribution over many trials, centered on the prototype). The noise is modality-specific, with the short axis of the ellipse aligned with the auditory axis indicating greater auditory precision. Similarly, for a visual “ga”, the features are encoded with the visual features more accurately encoded than the auditory features (dark blue ellipse labeled “V” with short axis of ellipse along the visual axis). The unisensory representations are integrated using Bayes’ rule to produce an integrated representation that is located between the unisensory components in the “da” region of representational space (light blue ellipse, “AV”). (**B**) With causal inference, participants recombine the integrated multisensory representation (*C* = 1, gray ellipse labeled “AV”) with the two-cause representation, assumed for speech to be the auditory representation (*C* = 2, gray ellipse labeled “A”). The weight given to each representation is determined by the likelihood for a common cause of the AV representation. The distribution of the final representation across trials is shown with the red ellipse labeled “CI”. (**C**) Repetitions with co-articulation increase the prior probability of a common cause. Black circles illustrate trials with fewer and more repetitions. (**D**) For each location in the representational space, we can use the natural statistics of audiovisual speech and the number of stimulus repetitions to calculate the likelihood of a common cause. For the locations resulting from AbaVga (black circles), a common cause is less likely with fewer repetitions (more red within black circle in left panel) and more likely with more repetitions (more blue within black circle in right panel). (**E**) This change in common cause probability results in the final causal inference representation landing in different regions of representational space (left and right panels). Applying a linear decision rule to the causal inference representation (necessary because of the categorical nature of speech perception) allows for percept prediction. (**F**) Predicted behavioral responses for fewer repetitions (left panel) and more repetitions (right panel) showing increased McGurk responses (“da”) with more repetitions.

To understand how a subtle stimulus difference—repeating a double-syllable *vs*. repeating a mono-syllable—results in a large perceptual effect, we turn to the framework of causal inference in multisensory perception^1–3,33^. Multisensory speech perception is conceptualized as four linked steps. The first two steps match those found in traditional Bayesian models: the auditory and visual speech information are encoded with modality-specific noise and integrated to generate a multisensory representation of the stimulus (Fig. 4A). The stimuli in our studies (“ba”, “da”, and “ga”) are mapped to a two-dimensional representational space, with locations reflecting the relative similarity of their auditory (*x*-axis) and visual (*y*-axis) features. This results in a tripartite map with “da” intermediate between “ba” and “ga”. For a McGurk stimulus consisting of auditory “ba” and visual “ga”, the integrated representation is the average of the component locations, weighted by precision, and lies in the “da” region of the space.

The observation that many participants do not report “da” when presented with the McGurk stimulus suggested the need for causal inference to supplement traditional Bayesian models (Fig. 4B). Rather than mandating integration of the auditory and visual representations, causal inference adds an additional step in which the likelihood of the integrated representation is assessed. If it is unlikely, perception is heavily weighted to the unisensory cue with the greatest reliability (for speech, this is the auditory representation). The final representation of the causal inference model is therefore a location in representational space intermediate between the auditory representation and integrated audiovisual representation. The higher the likelihood of a single talker, the closer the final representation is to the integrated representation.

For multisensory speech, there are many cues that can be used to assess the likelihood that the auditory and visual speech streams arise from a single talker. A particularly powerful cue is the temporal coincidence between visual mouth gestures and auditory speech features. In the McGurk effect, there is strong temporal synchrony between the mouth gesture “ga” and the auditory syllable “ba”. The temporal synchrony cue provides evidence for a single talker. This opposes the speech content cue, which provides evidence for separate talkers (because of the disparity between the “ga” mouth gesture and the auditory “ba”).

Under the causal inference model, additional repetitions with co-articulation provide accumulating evidence for a single talker (Fig. 4C). If only a single syllable is heard, the auditory and visual syllables could have been temporally coincident by chance. As more and more syllables are heard, the likelihood of chance coincidence decreases and subjects are more likely to assign the auditory and visual speech tokens to a single talker. There is behavioral evidence for such a benefit of audiovisual streams compared to isolated sounds^34,35^.

In the causal inference model, this is quantified as the likelihood that the encoded integration representation of the McGurk stimulus emanates from a single talker or two talkers (Fig. 4D). The speech content incongruity, which provides evidence for two talkers, stays the same with multiple repetitions while the evidence for a single talker provided by temporal synchrony, grows, resulting in a change in the perceived causal structure.

In the final step of the model, a linear classifier is used to generate a categorical percept from the causal inference representation (Fig. 4E). Initially, the likelihood of one *vs*. two talkers is similar, and the causal inference representation is intermediate between the unisensory auditory “ba” representation and the integrated representation, predicting a mixture of “ba” and “da” responses (Fig. 4F). As the repetitions accumulate, the likelihood of a single talker increases, and the causal inference representation moves closer to the integrated representation, resulting in a preponderance of “da” responses.

Our results are consistent with evidence from studies that study the effect of exposure to congruent or incongruent speech tokens on the McGurk effect^20,21^. Exposure to even brief periods of incongruent audiovisual speech lowers McGurk prevalence. This suggests that the probability of integration is influenced by recent experience with speech and that the probability of integration can be separated from the integration process, consistent with the tenets of causal inference. An interesting question for further research is how an incongruent contextual cue (provided by pre-exposure to incongruent speech) would interact with the temporal cue provided by repeated presentation of co-articulated McGurk syllables.

### Other factors contributing to the observed results

Experiment 2 demonstrated that stimulus repetition alone (without co-articulation) did not increase the prevalence of the McGurk effect. While it might be expected that gaining experience with the pairing of auditory “ba” and visual “ga” for a single talker might increase the likelihood of integration, we did not observe this in our data. This suggests that there is little learning in a paradigm with relatively few exposures. We also did not provide feedback; rewarding participants for reporting the McGurk percept could lead to different results. Experiment 3 found that talker familiarity (generated with brief exposure to a previously unknown talker) did not result in a change in McGurk prevalence. This is consistent with a previous report showing no effect of familiarity^32^, even for talkers who were personally familiar to the participant.

### Using Amazon Mechanical Turk for studying individual differences in speech perception

Because we wished to measure the effect of stimulus repetition without the confound of talker repetition, we used different talkers for each stimulus. This required a between-subjects design. To obtain the larger sample size required by these designs, data was collected using the Amazon Mechanical Turk (MTurk) online testing platform. Experiment 4 demonstrated the validity of this approach by showing no difference in McGurk effect for the same stimuli tested on-line and in-person.

## Conclusions

From a causal inference perspective, a major factor in integrating incongruent audiovisual stimuli is the relative strength of the temporal synchrony cue *vs*. the voice/lip correspondence cue. We have shown that syllable repetition with co-articulation can strengthen the evidence for a common cause, and therefore increases the tolerance to poor audiovisual correspondence. Mere repetition of a McGurk syllable, however, was insufficient to establish strong evidence for a common cause. We also tested the ability of a brief talker familiarization period to modulate the audiovisual correspondence but found no effect on integration. This result is in-line with previous work arguing little impact of familiarity on McGurk perception^32^ and mismatched face/voice gender on McGurk prevalence^36^. Taken together, the mere existence of seemingly relevant and detectable cue is not sufficient for it to be used in the causal inference decision (or, more precisely, not given a large relative weight in the causal inference decision). One intriguing possibility is that cues related informing *where* (spatial location), *when* (temporal synchrony), and *what* (speech content) are used for the causal inference decision, but not cues related to *who* is doing the talking (talker identity, talker gender).

## Method

All experiments were performed in accordance with relevant guidelines and regulations and was approved by the Rice University Institutional Review Board. All participants provided informed consent. For the online testing conducted for experiments 1–4, participants were recruited using Amazon Mechanical Turk^11,37,38^. For the in-person testing conducted for experiment 4, participants were recruited from the Rice University community.

### General Procedure

Both online and in-person experiments followed the same template. First, participants completed a brief demographics questionnaire and affirmed normal or corrected-to-normal hearing and vision. Second, a congruent audiovisual syllable video was displayed and participants were instructed to adjust the volume and window size until they could see and hear the video clearly. Third, task instructions were presented on-screen: “You will see videos and hear audio clips of a person saying syllables. Please watch the screen at all times. After each syllable, press a button to indicate what the person said. If you are not sure, take your best guess.” Fourth, participants were presented with a sequence of experimental stimuli. After each stimulus presentation, participants selected the response that best matched their percept from three on-screen choices: “ba”, “da/tha”, or “ga”. Experiments were self-paced, and no feedback was given.

These three choices were based on a previous experiment using McGurk stimuli with an open choice response format^11^. In this experiment, the vast majority of responses consisted of either “ba”, “ga”, “da” or “tha”. The advantage of a forced choice design is that it greatly increases the efficiency of data collection, as participants do not need to type a response following each and every trial, and the experimenter does not need to code every possible response variant (*e*.*g*. “da”, “thah”, “d-tha”, *etc*.).

### Experiment 1

#### Participants

Participants (*N* = 76; 32 Female; mean age = 32 ± 9 years, range = 18 to 65 years) were recruited using the Amazon Mechanical Turk platform. Participants performed the experiment on their own device in a location of their choice by signing into the MTurk website. Since speech stimuli are sensitive to the quality of the stimulus presentation, software checks were used to ensure that participants used a supported web browser. We tested the supported web browsers on a variety of hardware/software platforms to ensure that the stimuli displayed properly.

#### Stimuli

The control stimuli consisted of three highly-discriminable congruent audiovisual syllables (“ba”, “da”, and “ga”) produced by a female talker^11^. The experimental stimuli consisted of five variants of the McGurk incongruent syllable pair of auditory “ba” dubbed over visual “ga” (AbaVga). One stimulus was taken from a previous study (Stimulus 2.5 from^11^) and the remaining four consisted of a two-by-two design with two talkers and two repetition counts (6 or 1). The 6-repetition stimulus consisted of the talker repeating the double syllable “ba-ba” or “ga-ga” three times and the 1-repetition stimulus was a single repetition selected from the 6-repetition stimulus. The stimuli are freely available at http://openwetware.org/wiki/Beauchamp:DataSharing.

#### Procedure

Participants completed the experiment in one of two counterbalancing groups, each of which viewed the 1-repetition stimulus from one talker and the 6-repetition stimulus from the other talker. Both counterbalancing groups viewed the previously created McGurk stimulus. Participants viewed their group’s McGurk stimuli 10 times each (30 trials), randomly interleaved with audiovisual congruent stimuli (presented 3 or 4 times each, randomly determined) for a total of 40 trials.

### Experiment 2

#### Participants

Participants (*N* = 40; 22 Female; mean age 31 ± 8 years, range = 18 to 55) were recruited from Amazon Mechanical Turk.

#### Stimuli

Stimuli consisted of the three congruent audiovisual syllables “ba”, “da”, and “ga” from experiment 1, and 8 McGurk stimuli in a two talker by four repetition level (1, 2, 3 or 6 repetitions) design. The base stimuli consisted of a single-syllable McGurk stimuli (AbaVga) recorded from 8 different talkers (4 male, 4 female) in a previous study (stimuli 2.1 through 2.8 in^11^). The single-syllable stimuli from each talker were concatenated to create 2, 3, or 6 total syllable utterances using the *morph* option in Final Cut Pro to smooth the transition between the repetitions. To minimize carry-over effects, different talkers were used for each repetition level (two for each level).

#### Procedure

Participants viewed each of the 8 McGurk stimuli 10 times (80 total trials), randomly interleaved with the 3 control stimuli (viewed 3 or 4 times each, 10 total trials).

### Experiment 3

#### Participants

Participants (*N* = 60; 23 Female; mean age 32 ± 8 years, range = 21 to 69) were recruited from Amazon Mechanical Turk.

#### Stimuli

There were three types of stimuli. The familiarization stimuli consisted of congruent audiovisual stimuli (“ba”, “da” and “ga”) from two female talkers. The McGurk stimuli (AbaVga) were created from each talker’s congruent stimuli. Control stimuli were the congruent audiovisual stimuli (“ba”, “da”, and “ga”) from experiments 1 and 2.

#### Procedure

Participants completed the experiment in one of two counterbalancing groups, each of which were familiarized to one talker by viewing that talker’s congruent audiovisual stimuli (10 repetitions each, randomly interleaved, 30 trials total). After this familiarization phase, participants viewed McGurk stimuli for both talkers (10 trials of each), randomly interleaved with audiovisual congruent stimuli (presented 3 or 4 times each, randomly determined).

### Experiment 4

#### Participants

Online participants (*N* = 160; 76 Female) were recruited from Amazon Mechanical Turk; data from these subjects was previously reported^11^. In-person participants (*N* = 60; 35 Female) were recruited from Rice University.

#### Stimuli

Audiovisual stimuli consisted of McGurk stimuli (AbaVga) from eight different talkers and congruent syllables (AbaVba, AdaVda, AgaVga) from a ninth talker. Unisensory stimuli consisted of auditory syllables (Aba, Ada, Aga) and visual syllables (Vba, Vda, Vga) recorded from the same eight talkers used for the McGurk stimuli.

#### Procedure

The same stimuli were presented to online and in-person participants and the same software interface was used to present stimuli and record responses. For online testing, one group of subjects (*N* = 117) viewed only audiovisual syllables and another group (*N* = 50) viewed only unisensory stimuli. Seven participants were in both groups. For the first group, McGurk stimuli were presented 10 times each, randomly interleaved with audiovisual congruent stimuli (presented 3 or 4 times each, randomly determined) for a total of 40 trials; all other experimental details matched experiment 1. For the second group, 96 randomly interleaved unisensory stimuli were presented (8 talkers x 3 syllables x 2 modalities x 2 repetitions per stimuli). These data have previously been reported as experiments 2 and experiment 3, respectively^11^.

In-person testing was conducted with participants seated approximately 60 cm from a laptop (15” MacBook Pro) connected to wired headphones. In the first testing block, the 96 unisensory trials were presented; in the second testing block, the 40 audiovisual trials were presented.

### Experiments 1–4: Data analysis

For each subject, the responses to all congruent stimuli were combined into a single overall accuracy percentage. For McGurk stimuli, the percentage of “da/tha” responses was used as the estimate of McGurk prevalence for that stimulus and participant. We adopted this approach because there was only a very small fraction of “ga” (visual) responses, so that analyzing the visual-only responses did not produce informative results, consistent with previous results^11^. To model the effects of the experimental manipulations on McGurk prevalence, generalized linear mixed effects (GLMM) models were constructed in R (using function *glmer*, with family set to *binomial*) with the *lme4* package^39,40^. To convert the behavioral data into count format, the data was coded as the frequency of “da/tha” responses and frequency of not “da/tha” responses.

The baseline model included random effects of participant and stimulus; a fixed effect for counterbalancing group was included for experiments 1 and 3. The second model consisted of the baseline model with an additional fixed effect for the experimental manipulation. In experiment 1, the experimental manipulation of “repetition with co-articulation” was coded a categorical variable (repetition or not). In experiment 2, the manipulation of “number of repetitions (without co-articulation)” was coded as a scalar (1, 2, 3, or 6). In experiment 3, the manipulation of “talker familiarity” was coded as a categorical variable (familiar or not). In experiment 4, the manipulation of “testing mechanism” was coded as a categorical variable (online or in-person).

To choose the best model, we used Bayesian Information Criterion (BIC). Lower BIC values correspond to a better fit of the data to the model. BIC includes a penalty term such that, given equal fit to the data, the model with fewer parameters is preferred to the model with more parameters. The difference in BIC (ΔBIC) is a measure of the relative fit of the two models given the data. Differences of 10 or higher are considered decisive in favor of the model with the lower BIC^41^. For the nested models considered in this paper, even small BIC differences in favor of the baseline model (baseline model BIC < complex model BIC) are interpreted as supportive of the baseline model because the maximum BIC difference in favor of the baseline model is limited by the number of observations. We use this direct model comparison technique because it can determine which of the two models is best given the data, rather than merely *failing to reject* a null model of no difference between the groups. To provide an alternative to assess the statistical evidence for an experimental manipulation, we also provide *p*-values for the fixed-effect of interest in each experiment, corresponding to the null hypothesis that the true odds-ratio is 1.

## Data Availability

The datasets generated during and/or analysed during the current study are available from the corresponding author on reasonable request.
